# Risk factors for colonization with multiple species of extended-spectrum beta-lactamase producing Enterobacterales: a case-case–control study

**DOI:** 10.1186/s13756-021-01018-2

**Published:** 2021-10-24

**Authors:** Isabelle Vock, Lisandra Aguilar-Bultet, Adrian Egli, Pranita D. Tamma, Sarah Tschudin-Sutter

**Affiliations:** 1 Division of Infectious Diseases and Hospital Epidemiology, University Hospital Basel, Petersgraben 4, 4031 Basel, Switzerland; 2 Division of Bacteriology and Mycology, University Hospital Basel, University of Basel, Basel, Switzerland; 3 Applied Microbiology Research, Department of Biomedicine, University of Basel, Basel, Switzerland; 4 Department of Pediatrics, Johns Hopkins University School of Medicine, Baltimore, MD USA; 5 Department of Clinical Research, University Hospital Basel, Basel, Switzerland

**Keywords:** Extended-spectrum beta-lactamase producing Enterobacterales, ESBL, Co-colonization

## Abstract

**Background:**

Approximately 11% of patients colonized with extended-spectrum beta-lactamase producing Enterobacterales (ESBL-PE) are colonized with more than one ESBL-producing species. We investigated risk factors associated with colonization with multiple ESBL-PE species.

**Methods:**

We performed a case-case–control study at the University Hospital Basel, Switzerland, including hospitalized patients colonized with ESBL-PE between 01/2008 and 12/2018. Patients colonized with multiple species of ESBL-PE during the same hospitalization were assigned to group 1. Group 2 consisted of patients with ESBL-PE and a newly acquired ESBL-PE-species identified during subsequent hospitalization. Controls (i.e., group 3) were patients with only one species of ESBL-PE identified over multiple hospitalizations. Controls were frequency-matched 3:1 to group 2 cases according to time-at-risk (i.e., days between ESBL-PE detection during first and subsequent hospitalizations) to standardize the duration of colonization. ESBL was identified with phenotypic assay and the presence of ESBL genes was confirmed by whole genome sequencing.

**Results:**

Among 1559 inpatients, 154 cases met eligibility criteria (67 in group 1, 22 in group 2, 65 in group 3). International travel within the previous 12 months (OR 12.57, 95% CI 3.48–45.45, p < 0.001) and antibiotic exposure within the previous 3 months (OR 2.96, 95% CI 1.37–6.41, p = 0.006) were independently associated with co-colonization with multiple ESBL-PE species. Admission from another acute-care facility was the only predictor of replacement of one ESBL-PE species with another during subsequent hospitalizations (OR 6.02, 95% CI 1.15–31.49, p = 0.003).

**Conclusion:**

These findings point to strain-related factors being the main drivers of co-colonization with different ESBL-PE and may support stratification of infection prevention and control measures according to ESBL-PE species/strains.

**Supplementary Information:**

The online version contains supplementary material available at 10.1186/s13756-021-01018-2.

## Background

Over the past decade, the incidence of extended-spectrum beta-lactamase producing Enterobacterales (ESBL-PE), in particular *Escherichia coli* and *Klebsiella pneumoniae*, has increased rapidly worldwide [1], resulting in their classification as serious and critical threats by public health authorities, such as the Centers for Disease Prevention and Control (CDC) and the World Health Organization (WHO) [2, 3]. Asymptomatic colonization has been shown to be a primary risk factor for subsequent ESBL-PE infections [4–6]. Several patient-related characteristics have become recognized as risk factors for colonization with ESBL-PE, establishing the foundation for the development of prediction tools [7–14].

Previous investigations have demonstrated that colonization with multiple species of ESBL-PE occur in up to 11% of patients colonized with ESBL-PE [8, 15, 16]. However, data on risk factors and outcomes of co-colonization with different ESBL-PE species are incomplete. Whether patients colonized or infected with multiple ESBL-PE species over time acquire a new ESBL-PE strain or whether their incident ESBL-PE species horizontally transferred plasmids harboring ESBL genes to other colonizing Enterobacterales species remains largely unknown. Understanding this fundamental question will provide insights on the evolving epidemic of ESBL-PE and will inform future infection prevention and antibiotic stewardship interventions to interrupt this pathway. We sought to evaluate patient-related characteristics and exposures associated with colonization with multiple rather than single ESBL-PE species and to identify associated ESBL-gene types to gain further epidemiological insights.

## Methods

### Setting and participants

**Fig. 1:**
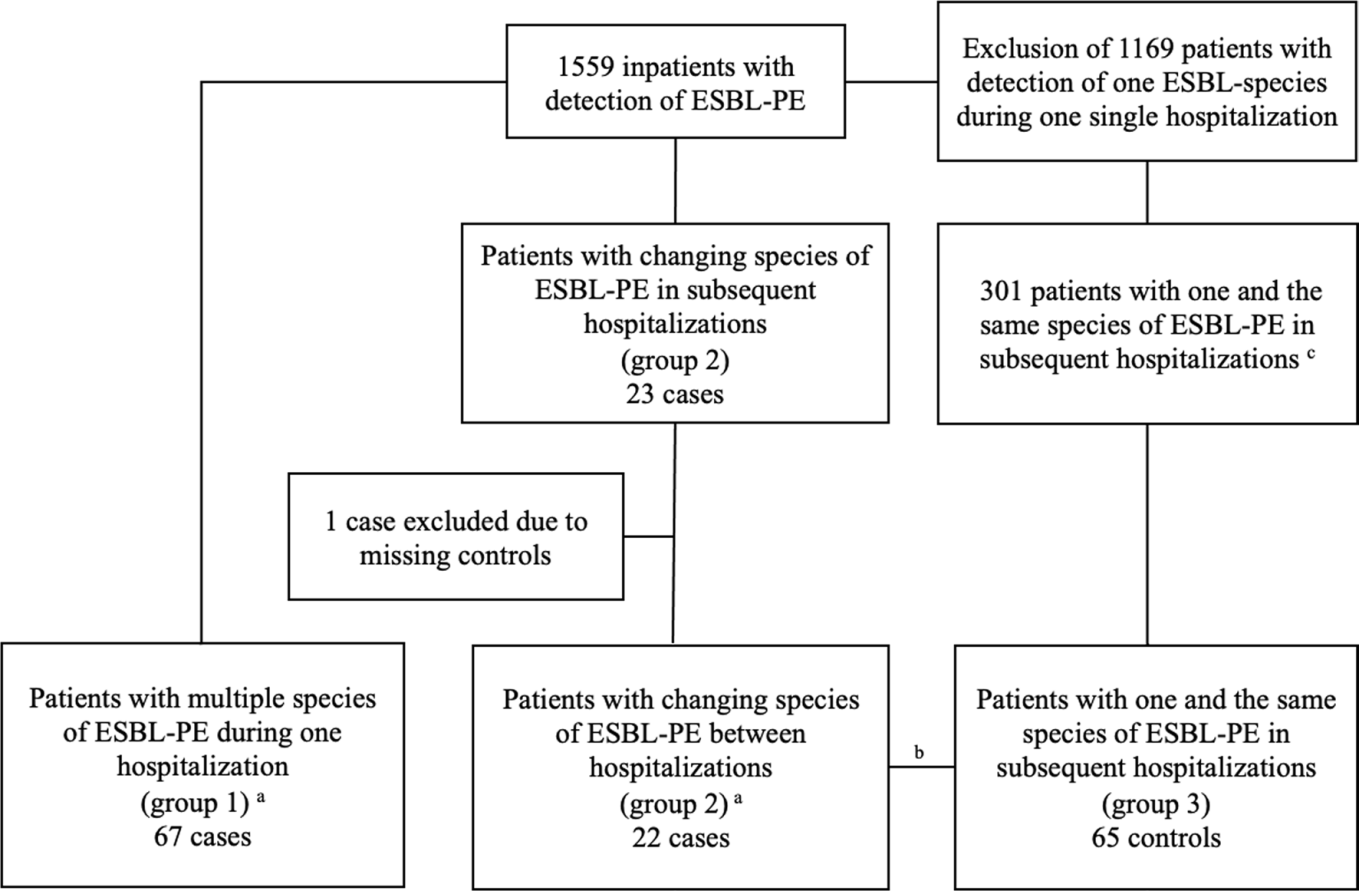
Flowchart of the selection of cases and controls, January 2008–December 2018. ^a^Four patients were eligible for both groups, hence were included in each. ^b^1:3 ratio matching for patients of group 2. Due to one missing control, one case was matched 1:2, resulting in 65 control patients. ^c^after exclusion of patients of group 1 and 2 as well as patients without a consecutive hospitalization with detection of the same ESBL-PE. ESBL-PE: extended-spectrum beta-lactamase-producing Enterobacterales

We conducted a retrospective observational case-case–control study at the University Hospital Basel (USB), a tertiary care academic centre admitting over 30,000 patients per year. Patients aged ≥ 18 years and hospitalized from January 2008 until December 2018 with ESBL-PE (as defined below) identified in any clinical or surveillance culture during their hospital-stay were eligible for study inclusion. Herein, “colonization” refers to identification of an ESBL-PE in either a clinical or surveillance culture. Eligible patients and bacterial strains were identified by systematically screening the electronic database of the Clinical Bacteriology and Mycology Laboratory. Strains were accessed via the Clinical Bacteriology and Mycology strain collection. Patients were assigned to the following groups (Fig. 1):

*Group 1*: Patients colonized with multiple species of ESBL-PE (*Escherichia coli, Klebsiella* species*, Proteus* spp*., Citrobacter* spp*., Morganella morganii, Serratia marcescens*, *Enterobacter* spp*., Pantoea* spp.) within the same hospitalization.

*Group 2*: Patients colonized with ESBL-PE in a first hospitalization and consecutive detection of a different species of ESBL-PE within a following hospitalization during the study period.

*Group 3*: Patients colonized with only one species of ESBL-PE across multiple hospitalizations during the study period.

Matching between group 2 and group 3 (i.e., the control group) was performed according to time at risk. Time at risk was defined as the number of days between detection of ESBL-PE during the first and subsequent hospitalizations, and was allowed to deviate by a maximum of 25% between cases and controls. These matching criteria were selected to ensure an equitable observation period by providing control patients with the “opportunity” to be colonized with different ESBL-PE species. Three controls were included for each group 2 patient. Risk factors predisposing to case or control status were determined. Additionally, the incidence of ESBL-PE infections and the distribution of ESBL-genes in each group were compared.

This study was approved by the local ethics committee (EKNZ – 2017 00100) and adhered to the Strengthening the Reporting of Observational Studies in Epidemiology (STROBE) guidelines for reporting of observational studies [17].

### Data collection

Clinical data were manually extracted from patient’s electronic medical records and entered into a secure REDCap database [18]. Missing data were categorized as negative risk factor. The following variables were collected based on biologic plausibility or their identification in the published literature [19–22] at the time of each case or control specimen collection date: (1) demographic data; (2) admission and discharge dates and destinations; (3) hospitalization within the previous 12 months, with at least one overnight stay in an acute care or long-term care facility; (4) travel, residence, or hospitalization outside of Switzerland with at least one overnight stay abroad within the previous 12 months; (5) microbiological data (including bacterial genus and species, antibiotic susceptibility data, ESBL status, source of culture, clinical versus surveillance culture, previous history of ESBL-PE colonization or infection); (6) underlying medical conditions on hospital admission based on the Charlson Comorbidity Index (CCI); (7) active open wounds (i.e., diabetic ulcers, decubitus ulcers, or other draining wounds); (8) surgical interventions within six months; (9) indwelling vascular hardware in place for at least 7 days; (10) urinary catheterization (i.e., Foley catheter, intermittent urethral catheterization, ureteral catheters, suprapubic catheterization) within 30 days; (11) history of solid organ or allogenic stem cell transplantation; (12) dialysis; (13) intravenous or oral antibiotic therapy within three months; (14) immunosuppressive therapy (i.e., corticosteroids, calcineurin-inhibitors, mTOR-inhibitors, cytostatics and monoclonal antibodies, or mycophenolates) within 12 months; (15) antacid medications (i.e., proton-pump inhibitors [PPI] or H2-antagonists) within 3 months.

### ESBL-PE identification

Stool surveillance specimens were plated onto selective chromogenic agar (chrom ID ESBL, bioMérieux, Marcy-l’Étoile, France). For clinical specimens, bacteria were identified either by MALDI-TOF mass spectrometry (Bruker Daltonics, Bremen, Germany) or by the Vitek 2™ System (bioMérieux, Durham, NC, USA). The Vitek 2™ System was used for susceptibility testing for all isolates. Non-susceptibility to cefpodoxime, ceftriaxone, ceftazidime, or aztreonam was used as a proxy for presumed ESBL production. Phenotypic confirmation of ESBL production was performed using Etest^®^ strips (bioMérieuex, Marcy-l’Etoile, France) or ROSCO disks (Rosco, Taastrup, Denmark). Antibiotic minimal inhibitory concentration (MICs) were interpreted according to EUCAST guidelines (www.eucast.org). Indeterminate results were further evaluated using the Eazyplex Superbug CPE panel (amplex, Gars-Bahnhof, Germany) which include the *bla*_CTX-M-1_ and *bla*_CTX-M-9_ gene groups. The *bla*_CTX-M-1_ and *bla*_CTX-M-9_ ESBL groups include the most common ESBL genes identified globally (e.g., *bla*_CTX-M-15_, *bla*_CTX-M-14_). If these genes were not present, isolates were considered ESBL negative [23].

### DNA extraction, library preparation and whole genome sequencing (WGS)

ESBL-PE isolates underwent WGS to identify ESBL genes and to evaluate relatedness of bacterial isolates. Bacteria were grown in blood-agar plates overnight (O/N) and whole bacterial DNA was extracted with the QIAamp DNA Mini kit (QIAGEN) in the QIAcube machine (QIAGEN), according to manufacturer conditions. Genomic libraries were prepared using Nextetra XT protocols (Illumina, San Diego) and WGS was performed using the NextSeq platform (Illumina, San Diego) (read length 2 × 150 bp). Quality control, filtering, and trimming raw sequencing data was performed with the fastp program v.0.20.0 [24]. Antimicrobial resistance genes were predicted directly from the pre-processed FASTQ paired-end reads using the ARIBA tool v.2.14.4 [25] against the ResFinder database [26]. Further classification of the beta-lactamase genes was performed according to the free access lists of the The Galileo AMR database (https://galileoamr.arcbio.com/mara/feature/list), the sequence annotation file of the Digital Multiplex Ligation Assay method validation (https://github.com/manutamminen/dmla), and after exhaustive literature searches.

### Statistical analyses

The Fisher’s exact test was used for comparisons of proportions of categorical variables. All continuous variables were found to be abnormally distributed after performing the Shapiro–Wilk test. Therefore, the Mann–Whitney U test was applied to determine medians and interquartile ranges. Univariable analysis with calculation of odds ratios was performed using logistic regression analysis for the non-matched comparisons of patients colonized with multiple ESBL-PE species (group 1) and the control group (group 3). Logistic regression using stepwise forward/backward regression as well as Akaike information criterion (AIC) was performed to identify risk factors independently associated with colonization with multiple ESBL-PE species. Conditional regression analysis was used for comparison of patients with a shift of ESBL-PE (group 2) and matching controls (group 3). *p*-values ≤ 0.05 were considered significant. STATA version 16.0 (StataCorp, College Station, TX) and R version 3.6.1 (R Foundation for Statistical Computing, Vienna, Austria) were used for statistical analyses.

## Results

### Characteristics of cases and controls

Among 1559 inpatients colonized with ESBL-PE during the study period, 154 patients met the eligibility criteria (Fig. 1). Group 1 consisted of 67 consecutive patients harbouring multiple ESBL-PE species during a single hospitalization; accounting for 4.3% of all ESBL carriers. Group 2 included 22 cases with new ESBL-PE species identified during subsequent hospitalizations and group 3 consisted of 65 patients with the same ESBL-PE identified during multiple hospitalizations.

Comparisons of baseline characteristics between the three groups are presented in Table 1. The majority of patients were hospitalized in medical wards. Patients were most frequently admitted from home and the distribution of patient age, sex, and burden of comorbidities were similar across groups. Patients belonging to group 1 more frequently reported travelling outside of Switzerland or being hospitalized abroad, and were more likely to be previously exposed to antibiotic treatment, as compared to patients belonging to groups 2 and 3. Group 1 patients were more commonly colonized rather than infected with ESBL-PE as compared to patients belonging to groups 2 and 3. *Escherichia coli* and *Klebsiella pneumoniae* were the most frequently detected species of ESBL-PE in each group (Table 1). Length of hospital stay, in hospital death and discharge destination were similar between the three groups (Table 1).

**Table 1:** A comparison of patient and microbial characteristics and clinical outcomes between three patient groups with extended-spectrum beta-lactamase-producing Enterobacterales (ESBL-PE) colonization

	Group 1^a^ N = 67		Group 2^a^ N = 22		Group 3^a^ N = 65		p-value
	n/median	% or IQR	n/median	% or IQR	n/median	% or IQR	
*Demogrophics*							
Age [years]	65	49–76	67.5	50–78	67	57–77	0.608
Female sex	35	52.2%	12	54.6%	32	49.2%	0.927
ICU Stay	22	32.8%	5	22.7%	20	30.8%	0.702
Admission from							0.265
Home	49	73.1%	15	68.2%	53	81.5%	
Other acute-care facility	13	19.4%	6	27.3%	6	9.2%	
Nursing-home	5	7.5%	1	4.6%	4	6.2%	
Unknown	0	0%	0	0%	2	3.1%	
*Exposures*							
Recent hospitalization^b^	49	73.1%	13	59.1%	42	64.6%	0.399
Including ICU stay	14	20.9%	2	9.1%	10	15.4%	0.466
History of stay outside of Switzerland^b^	25	37.3%	2	9.1%	3	4.6%	< 0.001
Europe	11	16.4%	1	4.6%	2	3.1%	
Asia	7	10.5%	0	0%	1	1.5%	
North America	1	1.5%	0	0%	0	0%	
South America	1	1.5%	0	0%	0	0%	
Africa	5	7.5%	1	4.6%	0	0%	
Hospitalisation abroad^b^	18	26.9%	1	4.6%	1	1.5%	< 0.001
Prior antibiotic therapy^h^	41	61.2%	9	40.9%	23	35.4%	0.010
Aminoglycosides	6	9.0%	1	4.6%	1	1.5%	
Carbapenems	8	12.0%	2	9.1%	4	6.2%	
1st and 2nd generation cephalosporins	1	1.5%	0	0%	1	1.5%	
3rd, 4th and 5th generation cephalosporins	12	17.9%	1	4.6%	2	3.1%	
Cotrimoxazole	11	16.4%	2	9.1%	5	7.7%	
Fosfomycin	0	0%	0	0%	2	3.1%	
Fluoroquinolones	10	14.9%	3	13.6%	3	4.6%	
Glycopeptides	3	4.5%	2	9.1%	2	3.1%	
Macrolides	2	3.0%	2	9.1%	2	3.1%	
Metronidazole	3	4.5%	3	13.6%	2	3.1%	
Penicillin	4	6.0%	0	0%	3	4.6%	
Penicillin-beta-lactamase-inhibitor	9	13.4%	4	18.2%	6	9.2%	
Piperacillin-tazobactam	11	16.4%	3	13.6%	9	13.9%	
Tetracycline	1	1.5%	0	0%	0	0%	
Other^g^	7	10.5%	1	4.55%	3	4.6%	
Duration of prior antibiotic therapy [days]	26	8–39	24	5–39	21	9–42	0.927
*Clinical characteristics*							
Charlson Comorbidity Index	2	0–3	2	1–3	2	1–3	0.945
Solid organ transplantation	4	6.0%	2	9.1%	5	7.7%	0.763
Allogenic stem cell transplantation	4	6.0%	0	0%	0	0%	0.117
Recent surgery^c^	21	31.3%	6	27.3%	22	33.9%	0.892
Indwelling vascular hardware^d^	4	6.0%	0	0%	2	3.1%	0.618
Urinary catheterization^e^	13	19.4%	4	18.2%	16	24.6%	0.799
Active open wounds^f^	6	9.0%	4	18.2%	8	12.3%	0.429
Dialysis	0	0%	0	0%	1	1.5%	0.565
Immunosuppressive therapy^b^	19	28.4%	4	18.2%	19	29.2%	0.643
Proton-pump inhibitor therapy^h^	40	59.7%	12	54.6%	36	55.4%	0.879
*Microbiological characteristics*							
ESBL – species							
*Escherichia coli*	61	91.0%	18	81.8%	55	84.6%	0.369
*Klebsiella pneumoniae*	57	85.1%	5	22.7%	9	13.9%	< 0.001
*Citrobacter *spp.	9	13.4%	2	9.1%	1	1.5%	
*Enterobacter cloacae*	7	10.5%	0	0%	0	0%	
*Proteus *spp.	3	4.5%	1	4.5%	0	0%	
*Klebsiella aerogenes*	2	3.0%	0	0%	0	0%	
*Klebsiella variicola*	2	3.0%	0	0%	0	0%	
Infection due to ESBL-PE^i^	18	26.9%	8	36.4%	31	47.7%	0.044
*Outcomes*							
Length of hospital stay [days]	20	10–34	11.5	5–22	15	5–29	0.091
In-hospital death	3	4.5%	0	0%	0	0%	0.284
Discharge destination							0.972
Home	35	54.7%	12	54.6%	36	55.4%	
Other acute-care facility	24	37.5%	9	40.9%	23	35.4%	
Long-term healthcare center	1	1.6%	0	0%	1	1.6%	
Nursing-home	4	6.3%	1	4.6%	3	4.6%	
Unknown	0	0%	0	0%	2	3.1%	

^a^Group 1 = patients with multiple species of ESBL-PE within one hospitalization, Group 2 = patients with shift of ESBL-PE species between hospitalizations, Group 3 = control patients with colonization of one species of ESBL-PE within different hospitalizations

^b^Within the previous 12 months

^c^Within the previous 6 months

^d^In place for at least 7 days prior to culture collection date

^e^Transurethral or suprapubic catheterization within the previous 30 days

^f^Diabetic ulcers, decubitus ulcers, or other draining wounds

^g^Daptomycin, Clindamycin, Rifampicin, Nitrofurantoin, Isoniazid, Ethambutol, Linezolid

^h^Within 3 months prior to the index sample

^i^Within the same hospitalization

**Table 2:** Univariable and multivariable analyses of potential predictors of colonization with multiple species of extended-spectrum beta-lactamase-producing Enterobacterales (ESBL-PE)-comparison of case group 1^a^ and control group 3^a^

	Univariable analyses			Multivariable analyses^b^		
	OR	95%CI	p-value	OR	95%CI	p-value
Age	0.99	0.97–1.01	0.204			
Female sex	0.89	0.45–1.75	0.730			
Admission from nursing-home / long-term healthcare	1.23	0.32–4.80	0.766			
Admission from other acute care facility	2.37	0.84–6.67	0.103			
Recent hospitalization^c^	1.50	0.71–3.13	0.291			
Recent ICU stay^c^	1.45	0.59–3.56	0.413			
Travel^c^	12.3	3.49–43.37	**< 0.001**	12.57	3.48–45.45	**< 0.001**
Hospitalization abroad^c^	23.51	3.03–182.21	**0.003**			
Charlson Comorbidity Index	0.96	0.81–1.14	0.632			
Solid organ transplantation	0.76	0.20–2.97	0.695			
Urinary catheterization^d^	0.74	0.32–1.69	0.470			
Vascular hardware^e^	2	0.35–11.31	0.433			
Recent surgery^f^	0.89	0.43–1.85	0.759			
Active open wounds^g^	0.70	0.23–2.14	0.533			
Prior antibiotic therapy^h^	2.88	1.42–5.84	**0.003**	2.96	1.37–6.41	**0.006**
Immunosuppressive therapy^c^	0.96	0.45–2.04	0.912			
Proton-pump inhibitor therapy^h^	1.19	0.60–2.38	0.616			

Bold represents statistically significant (p-values ≤ 0.05)

^a^Group 1 = patients with multiple species of ESBL-PE within one hospitalization, Group 3 = control patients with colonization of one species of ESBL-PE within different hospitalizations

^b^Multivariable analyses included the variables travel, hospitalization abroad, prior antibiotic therapy. Only variables identified by stepwise logistic regression using stepwise forward and backward selection as well as Akaike information criterion are presented in the table

^c^Within the past 12 months

^d^Transurethral or suprapubic within 30 days prior to index sample

^e^In place for at least 7 days prior to index sample

^f^Within the past 6 months

^g^Diabetic ulcers, decubitus ulcers, or other draining wounds

^h^Within 3 months prior to index sample

**Fig. 2:**
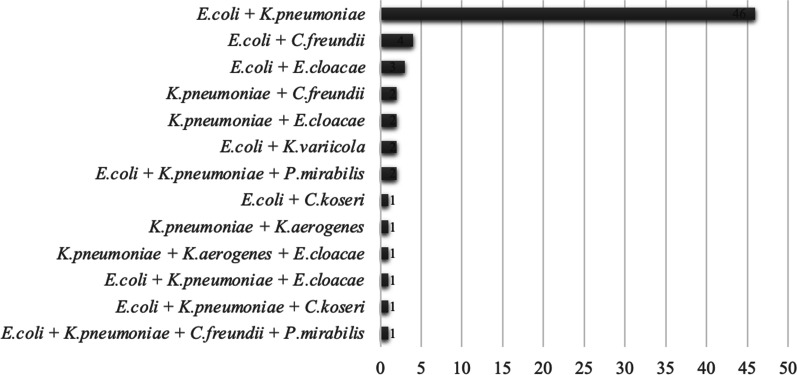
Frequency of combinations of extended-spectrum beta-lactamase-producing Enterobacterales identified during the same hospital stay

The distribution of different combinations of ESBL-PE within group 1 is shown in Fig. 2, the most prevalent combination being *E. coli* and *K. pneumoniae* (69%). Combinations always included either *E. coli*, *K. pneumoniae*, or both, along with a less common ESBL-PE species. Within group 2, the shift of species most frequently occurred from *E. coli* to *K. pneumoniae* (n = 11, 50%) (Additional file 1: Table S1).

### Comparisons across the three groups

Univariable comparisons between groups 1 and 3 revealed travel history, recent hospitalization abroad, and exposure to antibiotic therapy within the prior 3 months to be associated with simultaneous colonization with multiple ESBL-PE species. In an adjusted analysis, including these three variables, travel history and prior antibiotic therapy were independently associated with colonization of multiple ESBL-PE species (OR = 12.57; 95% CI 3.48–45.45, p-value < 0.001 and OR = 2.96; 95% CI 1.37–6.41, p-value = 0.006, respectively; Table 2).

Comparing groups 2 and 3, admission from another acute care facility was the only variable associated with an increased risk of shift of ESBL-PE species (OR 6.02; 95% CI 1.15–31.49, p-value: 0.033); Table 3.

**Table 3:** Univariable analyses of potential predictors of shift of extended-spectrum beta-lactamase-producing Enterobacterales (ESBL-PE) – comparison of case group 2^a^ and matched control group 3^a^

	Univariable analyses		
	OR	95%CI	p-value
Age	0.99	0.96–1.02	0.433
Female sex	0.82	0.30–2.23	0.701
Admission from nursing-home/long-term healthcare	0.75	0.08–6.71	0.797
Admission from other acute care facility	6.02	1.15–31.49	**0.033**
Recent hospitalization^b^	0.78	0.26–2.29	0.649
Recent ICU stay^b^	0.57	0.12–2.77	0.485
Travel^b^	2.01	0.28–15.43	0.477
Hospitalization abroad^b^	3.00	0.19–47.96	0.437
Charlson Comorbidity Index	0.98	0.77–1.26	0.934
Solid organ transplantation	1.26	0.20–8.03	0.809
Urinary catheterization^c^	0.68	0.20–2.30	0.536
Recent surgery^e^	0.74	0.26–2.11	0.579
Active open wounds^f^	1.59	0.43–5.82	0.483
Prior antibiotic therapy^g^	1.29	0.48–3.47	0.612
Immunosuppressive therapy^b^	0.54	0.16–1.84	0.322
Proton-pump inhibitor therapy^g^	0.97	0.33–2.87	0.963
Antibiotic use between hospitalizations	1.84	0.50–6.80	0.360
Duration of antibiotic therapy	1.00	0.99–1.00	0.295
Travel between hospitalizations	0.56	0.06–4.78	0.593
Hospitalization abroad	1.30	0.12–14.51	0.830

Bold represents statistically significant (p-values ≤ 0.05)

^a^Group 2 = patients with shift of ESBL-PE species between hospitalizations, Group 3 = control patients with colonization of one species of ESBL-PE within different hospitalizations

^b^Within the past 12 months

^c^Transurethral or suprapubic within 30 days prior to index sample

^d^In place for at least 7 days prior to index sample

^e^Within the past 6 months

^f^Diabetic ulcers, decubitus ulcers, or other draining wounds

^g^Within 3 months prior to index sample

### Comparisons between the distribution of ESBL-genes across the three groups

**Fig. 3:**
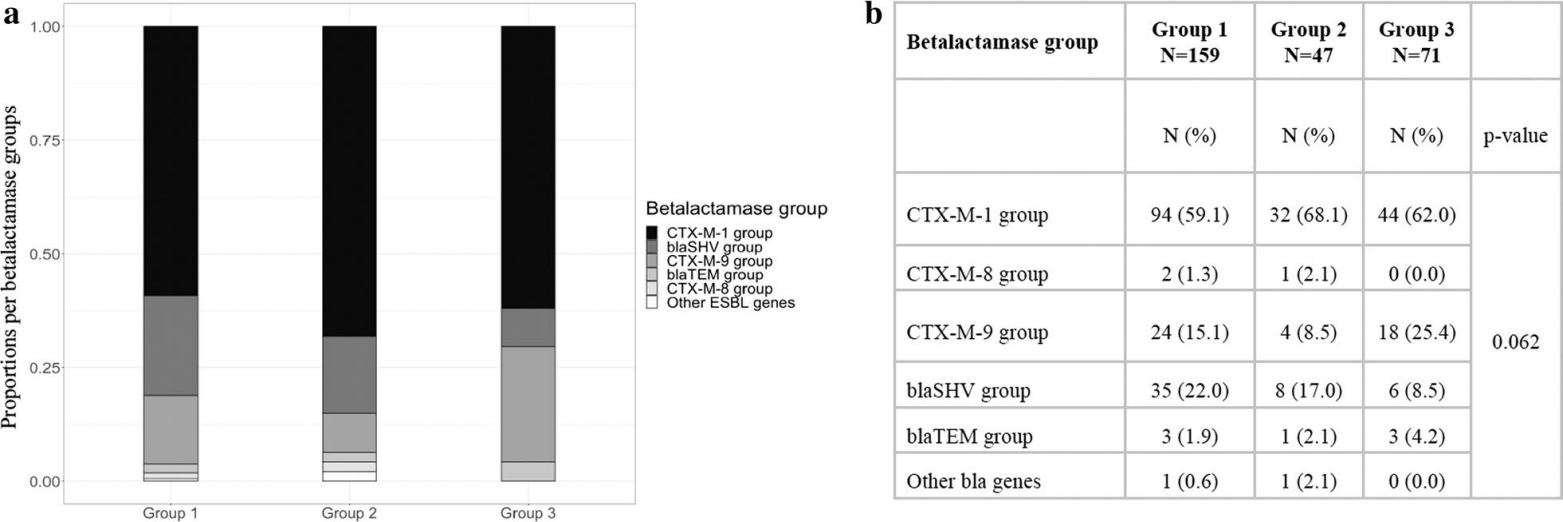
**a** Counts per betalactamase group. **b** Comparison of betalactamase groups of group 1, 2 and 3

ESBL-strains were available for 153 out of 154 patients (Fig. 1). 277 ESBL-genes were identified and belonged to the following groups: CTX-M-1 group (e.g., *bla*_CTX-M-15_, *bla*_CTX-M-1_, *bla*_CTX-M-3_), CTX-M-8 group (e.g. *bla*_CTX-M-8_), CTX-M-9 group (e.g., *bla*_CTX-M-14_ and *bla*_CTX-M-27_ (a single nucleotide variant of *bla*_CTX-M-14_)), and ESBL-variants of *bla*_SHV_ and *bla*_TEM_ (Fig. 3a). ESBL-gene-groups in case groups 1 and 2 were similarly distributed, and had higher proportions of *bla*_SHV_ genes and a lower proportion of *bla*_CTX-M-9_ group genes as compared to group 3 (Fig. 3b). Distribution of ESBL genes within the separate species are shown in the Additional file 1: Fig. S1.

## Discussion

We found that 4.3% of ESBL-PE carriers were simultaneously colonized with different species of ESBL-PE. Exposure to antibiotic therapy and travel abroad were associated with an increased likelihood of co-colonization with different ESBL-producing species rather than persistent colonization with a single species of ESBL-PE. Admission from another acute care facility was the only variable associated with an increased risk of shift of ESBL-PE species, while antibiotic exposure between hospitalization did not differ between patients with a shift of ESBL-PE species and between patients who remained colonized with a single species of ESBL-PE. Simultaneous or subsequent colonization with different species of ESBL-PE appears to result from either de novo acquisition of ESBL-PE strains or by transmission of ESBL-encoding genes and mobile genetic elements to colonizing non-ESBL-PE strains. The former potentially related to lapses in infection control practices and the later potentially facilitated by antibiotic selection pressure. Our findings support a strong role for the acquisition of novel strains in settings with differing ESBL-PE epidemiology (such as differing geographical regions or healthcare facilities) and suggest that antibiotic selection pressure may facilitate initial colonization, but seems less likely to induce transmission of ESBL-genes to other colonizing Enterobacterales.

Travel to high-endemic areas such as India or South East Asia is a well established risk factor for colonization with ESBL-PE [11], yet co-colonization with different species of ESBL-PE in travelers is presumably low (6.1% among 633 travelers from the Netherlands [11] and 8.6% among travelers from Germany [27] returning with ESBL-PE colonization). The higher frequency of ESBL- *E. coli*-colonization in returning travelers compared to colonization with other species of ESBL-PE (mainly *K. pneumoniae*) points to important differences in the epidemiology of these two species of Enterobacterales; *E. coli* being more likely related to community-acquisition and *K. pneumoniae* and other ESBL-PE species more likely related to healthcare-associated transmission [28, 29]. Hospitalization abroad did not remain significant in our adjusted analysis as a risk factor for colonization with multiple species of ESBL-PE, likely because of its collinearity with a history of stay outside of Switzerland. Yet, our findings of associations between antibiotic exposures and admission from other healthcare facilities with co-colonization support increased exposure to healthcare services being related with the risk of acquisition of non-*E. coli* ESBL-PE.

In addition to exposure to settings with differing ESBL-PE epidemiology, antibiotic therapy applying selective pressure has been indicated as a risk factor for colonization with ESBL-PE in various studies [30, 31] and it is not surprising that antibiotic use could foster an environment ripe for carriage of multiple ESBL-PE species. However, our findings did not demonstrate an association between receipt of antibiotic medication between hospitalizations and a shift in species of ESBL-PE. Given the high frequency of antibiotic use, a shift in ESBL-species would likely occur more often, if antibiotic pressure was an important driver of transmission of ESBL-genes within a host.

Across all three case groups investigated in this study, ESBL-genes from the group CTX-M-1 was the most predominant, which corresponds with it being the most widespread group worldwide [32, 33]. Additionally, ESBL-gene-groups in case groups 1 and 2 were similarly distributed, and had higher proportions of SHV genes and a lower proportion of CTX-M-9 group genes as compared to group 3. This difference derives may be related to the higher proportions of *K. pneumoniae* and from the co-existence of CTX-M-1 and SHV genes in 34.2% and 27.2% of isolates in groups 1 and 2, respectively; while in group 3 only 3.1% of the isolates harboured both gene-groups simultaenously. The presence of the CTX-M-9 group as the second most common EBSL genes in group 3 corresponds to its global prominence [32, 33].

Our study has some important limitations. The retrospective single center study design limits the generalizability of findings to other settings. Travel history is not systematically assessed at hospital admission in our institution and performed mostly in patients with clinical suspicion of travel-acquired infections, patients being repatriated, or patients with a background of migration and its inclusion in our study introduces information bias. A certain number of positive travel histories might have been missed, potentially leading to an overestimation of travel as a risk factor. The limited sample size might have led to our study being underpowered to detect additional risk factors associated with multiple ESBL-PE colonization. Our study occurred in a low ESBL-endemic setting, furthermore a global consensus on active surveillance methods is lacking and our systematic screening strategies for ESBL-PE may vary from other national and international institutions, hence further research is needed to evaluate these findings in regions with a higher prevalence of ESBL-PE.

## Conclusions

Co-colonization with different species of ESBL-PE is infrequent and likely to derive from exposure to settings with a differing ESBL-PE epidemiology, as may be encountered in other geographical regions and healthcare settings, further promoted by antibiotic exposure exerting selective colonization-pressure. These results point to specific ESBL-PE strains being the main driver of ongoing ESBL-transmission rather than ongoing host transmission of mobile genetic elements. These results also support the dissemination of ESBL-PE to non-ESBL-PE and support stratification of infection prevention and control measures according to ESBL-PE species/strains. ESBL surveillance frameworks should address potential co-colonization especially in patients with a history of travel abroad or hospitalization at different institutions.

## Supplementary Information

**Additional file 1.** **Supplementary Table 1:** Shift of species within group 2. **Supplementary Figure 1:** Distribution of ESBL genes within separate species.

## Data Availability

The datasets used and analysed during the current study are available from the corresponding author on reasonable request.

## Abbreviations

AIC: Akaike information criterion
CCI: Charlson Comorbidity Index
CDC: Centers for Disease Prevention and Control
EKNZ: Ethikkommission Nordwest- und Zentralschweiz (ethics commission of northern and central switzerland)
ESBL: Extended-spectrum beta-lactamase
ESBL-PE: Extended-spectrum beta-lactamase producing Enterobacterales
USB: University Hospital Basel
WGS: Whole genome sequencing
WHO: World Health Organisation
