# Supplementary material for: Risk factors for colonization with multiple species of extended-spectrum beta-lactamase producing Enterobacterales: a case-case–control study
Source: Antimicrob Resist Infect Control. 2021 Oct 24;10:153. doi: 10.1186/s13756-021-01018-2 (PMC8543947; doi:10.1186/s13756-021-01018-2)
Supplement: Supplementary file 1 — Additional file 1. Supplementary Table 1: Shift of species within group 2. Supplementary Figure 1: Distribution of ESBL genes within separate species. [file 13756_2021_1018_MOESM1_ESM.docx]

**Supplemental materials**

| **Supplementary Table 1** Shift of species of group 2 (N=22) | | |
| --- | --- | --- |
| **Species of 1^st^ hospitalisation** | **New species 2^nd^ hospitalisation** | **N (%)** |
| *Escherichia coli* | *Klebsiella pneumoniae* | 11 (50%) |
| *Escherichia coli* | *Enterobacter cloacae* | 2 (9.1%) |
| *Klebsiella pneumoniae* | *Escherichia coli* | 2 (9.1%) |
| *Escherichia coli*  *Klebsiella pneumoniae* | *Enterobacter cloacae* | 1 (4.5%) |
| *Klebsiella pneumoniae*  *Citrobacter freundii* | *Enterobacter cloacae* | 1 (4.5%) |
| *Proteus vulgaris* | *Escherichia coli* | 1 (4.5%) |
| *Escherichia coli* | *Citrobacter farmeri* | 1 (4.5%) |
| *Escherichia coli*  *Klebsiella pneumoniae* | *Enterobacter cloacae*  *Pantoea species* | 1 (4.5%) |
| *Citrobacter freundii*  *Escherichia coli* | *Enterobacter cloacae* | 1 (4.5%) |
| *Escherichia coli* | *Citrobacter koseri* | 1 (4.5%) |

Supplementary Figure 1

Supplementary Figure 1. Distribution of ESBL genes within each species of extended-spectrum beta-lactamase-producing Enterobacterales
